# S04 Sports Club for Health (SCforH) approach: evidence on importance and examples of implementation activities

**DOI:** 10.1093/eurpub/ckac093.016

**Published:** 2022-08-29

**Authors:** Željko Pedišić, Sylvia Titze

**Affiliations:** 1 Victoria University, Institute for Health and Sport, Melbourne, Australia; 2 University of Graz, Institute of Human Movement Science, Sport and Health, Graz, Austria

**Keywords:** sports club, implementation, adherence, dissemination, online learning

## Abstract

There are more than 60 million sports club members in the EU, which makes sports clubs an organizational settings with a huge potential for health promotion. The Sports Club for Health (SCforH) initiative started in 2008, to assist sports clubs in promoting health-enhancing physical activity. In support of this endeavour, SCforH guidelines were issued in 2011 and updated in 2017. The SCforH initiative has been supported by three large European Union grants and included partner organisations from 18 countries. In 2013, the Council of the European Union proposed the national implementation of SCforH guidelines as one of the 23 indicators for evaluation of HEPA levels and policies in the European Union member states. Recently, the European Commission recognised the SCforH initiative as a ‘success story' and an example of good practice. The aim of this symposium is to summarize recent evidence relevant to the promotion of health-enhancing physical activity through sports clubs in the EU and to discuss about future directions and challenges for EU public health efforts in this area. The symposium will include five 10-minute presentations on: [i] the findings of an updated systematic review on health benefits of different sport disciplines; [ii] Hungarian implementation of a SCforH intervention; [iii] the effectiveness of a coordinated action between the healthcare sector and local sports clubs to promote physical activity; [iv]; tracking participants in Jackpot.fit program?an Austrian sport promotion initiative; and [v] the development of a SCforH online learning tool. The symposium program will also include: Introduction by Chair (5 minutes), 25 minutes of Q&A, and conclusion by Discussant (10 minutes).

Discussant: Prof Nanette Mutrie, Moray House School of Education and Sport, ISPEHS research cluster, University of Edinburgh, United Kingdom - nanette.mutrie@ed.ac.uk

